# Metal Oxide Mediated Extracellular NADPH Regeneration Improves Ethanol Production by Engineered *Synechocystis* sp. PCC 6803

**DOI:** 10.3389/fbioe.2019.00148

**Published:** 2019-06-19

**Authors:** Rajendran Velmurugan, Aran Incharoensakdi

**Affiliations:** ^1^Cyanobacterial Biotechnology Laboratory, Department of Biochemistry, Faculty of Science, Chulalongkorn University, Bangkok, Thailand; ^2^Academy of Science, Royal Society of Thailand, Bangkok, Thailand

**Keywords:** ethanol, *Synechocystis*, NADPH regeneration, metal oxides, light intensity, electron donor, redirection of carbon-flow

## Abstract

The ethanol synthesis pathway engineered *Synechocystis* sp. PCC 6803 (hereafter *Synechocystis*) was used to investigate the influence of metal oxide mediated extracellular NADPH regeneration on ethanol synthesis. The *in-vitro* studies proved that the metal oxides have the potential to generate the NADPH in the presence of electron donor, the usual components of photoautotrophic growth conditions. When the NADPH regeneration was applied in *Synechocystsis*, the strain showed improved growth and ethanol production. This improved ethanol synthesis is attributed to the increased availability of NADPH to the ethanol synthesis pathway and redirection of closely related carbon metabolism into the ethanol synthesis. Under optimized light intensity and NADP addition, the maximum ethanol production of 5,100 mg/L was observed in MgO mediated extracellular NADPH regeneration after 25 days of cultivation, which is 2-fold higher than the control. This study indicates the feasibility of metal oxide mediated extracellular NADPH regeneration of *Synechocystis* to increase the production of ethanol.

## Introduction

To replace the fossil fuels, extensive studies have been focused on developing efficient technique for the production of ethanol from cyanobacteria (Gao et al., 2012; Velmurugan and Incharoensakdi, 2016; Liang et al., 2018). The incorporation of ethanol synthesis pathway from various microorganisms into cyanobacteria has already been demonstrated as a route of direct carbon fixation for ethanol production (Gao et al., 2012). However, other advancements in carbon fixation, metabolic flux, and nutrient supplementation are also reported for the betterment of the productivity (Nozzi et al., 2013; Liang et al., 2018). As a part of process improvement, engineering of the gene *zwf* which is responsible for NADPH regeneration has been demonstrated resulting in an improvement of cyanobacterial growth and ethanol production (Choi and Park, 2016). Generally, the regeneration of cofactor (NADPH/NADH) has been performed in the presence of oxidoreductases (van der Donk and Zhao, 2003) such as alcohol dehydrogenase (Ma et al., 2017), formate dehydrogenase (Yamamoto et al., 2005), glucose dehydrogenase (Wong et al., 1985), and phosphite dehydrogenase (Johannes et al., 2007); as well as redox molecules (Wong et al., 1985; Yamamoto et al., 2005); and light (Nakamura and Yamanaka, 2002; Ma et al., 2017). To improve the ethanol synthesis, it is also important that the NADPH has to be regenerated simultaneously during enzyme catalysis. Recently, the NADH regeneration was performed in the presence of metal oxides such as carbon containing TiO_2_ and P-doped TiO_2_ and electron mediator such as Ru(bpy)^2+^ or Zn(TMPyP)^4+^ or methyl viologen (Jiang et al., 2005; Shi et al., 2006). However, these components have disadvantages such as excitation of TiO_2_ upon ultraviolet light, toxicity, contamination of MV^2+^ and unstable structural properties of Ru(bpy)^2+^ and Zn(TMPyP)^4+^ (Grimes and Drueckhammer, 1993; Asahi et al., 2001). A system that includes an efficient electron transfer with cellular compatibility can be a feasible NADPH generation process. The EDTA and citrate, which are also components of growth media for cyanobacteria, have the characteristics to chelate the metal oxides and act as an electron donor in various reactions (Motekaitis et al., 1980; Jones et al., 2015). Development of a chemical method that exhibits NADPH generation under normal photoautotrophic growth conditions can be a microbial friendly process. The use of such growth conditions for NADPH regeneration can be a compatible process. Accordingly, the NADPH regeneration was performed for ethanol synthesis pathway engineered *Synechocystis* in the presence of metal oxides such as Fe_2_O_3_ and MgO. The influences of each factor such as electron donor, mediator and exogenous NADP were determined by *in-vitro* experiments.

## Materials and Methods

### Materials

The metal oxides such as Fe_2_O_3_ (Himedia Laboratories Ltd.) and MgO (QReC) were commercial products and the average sizes of the particle were 96 and 470 nm, respectively (Velmurugan and Incharoensakdi, 2016). The NADP, NADPH, NADP/NADPH assay kit were purchased from Sigma Aldrich. All the standard chemicals used for high performance liquid chromatography (HPLC) analysis were the products of Sigma Aldrich and the mobile phase (sulfuric acid) was the product of QRec.

### Microorganism and Cultivation Conditions

The *pdc-adh* pathway engineered *Synechocystis* sp. PCC 6803 (Pasteur Institute, France) was propagated on BG-11 agar medium (Rippka et al., 1979). The expression vector pAPX was constructed by insertion of NADP^+^ dependent alcohol dehydrogenase (*adh*: GenBank accession number MK893427) from *Synechocystis* sp. PCC 6803 and pyruvate decarboxylase (*PDC1*: GenBank accession number MK868028) from *Saccharomyces cerevisiae* into pEERM vector under the control of *psbA1* promoter (Data S1; Englund et al., 2015; Velmurugan and Incharoensakdi, 2018). The engineered strain was cultivated in 250 mL Erlenmeyer flasks containing 100 mL BG-11 medium (pH 7.5) supplemented with 30 μg/mL chloramphenicol under continuous illumination of 100 μE/m^2^/s at 28 ± 1°C with atmospheric CO_2_ supply upon shaking at 160 rpm (Rippka et al., 1979; Zhu et al., 2017).

### *In*-*vitro* Studies on NADPH Regeneration

The effects of metal oxides (Fe_2_O_3_ and MgO), light, and electron mediator on NADPH regeneration were studied by adding NADP (100 μM) in BG-11 media and MilliQ water. To determine the individual effects (metal oxides, BG-11, Na_2_EDTA, and light), the control experiments were performed by varying the conditions such as BG-11 medium/MilliQ water and without/with light (100 μE/m^2^/s). The reaction was performed in a total volume of 5 mL at 28 ± 1°C under shaking at 160 rpm. After 3 h of incubation, the reaction mixture was centrifuged at 12,000 × g for 5 min and filtered through centrifuging 500 μL sample in filtration tube. The samples were immediately used for NADPH per total NADP measurement.

### NADPH Regeneration With *Synechocystis*

The effect of metal oxide (Fe_2_O_3_: 3.2 mg/L and MgO: 8 mg/L) mediated NADPH regeneration on biomass, chlorophyll *a*, and ethanol production was analyzed by varying the NADP concentration (50–250 μM). To analyse the effect of NADPH regeneration, separate control experiments (no metal oxides) were performed with NADPH addition. The effect of continuous light illumination on ethanol production was studied by growing the cells (initial OD≈0.06 at 730 nm) in Erlenmeyer flask (250 mL) containing 100 mL BG-11 (pH 7.2) medium under various light intensities (50–200 μE/m^2^/s). The flasks were incubated at 28 ± 1°C under atmospheric CO_2_ supply upon shaking at 160 rpm for 20 days.

The scaleup experiment was performed in a 5 L photo-bioreactor equipped with controlled atmospheric air supply and the valves for inoculation and temperature monitor. The cells were cultivated in BG-11 medium (pH 7.2) with the atmospheric air supplementation at the flow rate of 200 mL/min under light (100 μE/m^2^/s) up to 25 days.

After the cultivation, the cells were harvested after 20 days of cultivation by centrifugation at 6,000 × g for 10 min at room temperature. The supernatant was used for the analysis of extracellular products such as acetate, ethanol, pyruvate, and succinate. For the analysis of intracellular products, the harvested cells were vigorously mixed with 500 μL of 70% methanol by vortex mixer. The mixture was incubated for 2 h at room temperature and then centrifuged at 6,000 × g for 10 min at 4°C. The supernatant was collected and dried in a vacuum evaporator at 40°C. Pellet left after drying was dissolved and mixed thoroughly in 250 μL of water and 50 μL of chloroform followed by centrifugation at 6,000 × g for 10 min (Kanwal et al., 2014). The uppermost water phase (200 μL) was collected, and filtered through a 0.45 μm Millipore filter before the analysis of acetate, ethanol, pyruvate, and succinate by HPLC.

### Analytical Methods

The dry cell weight (DCW) was determined by drying the pellet of centrifuged culture in an oven (Heratherm OGS750, Thermoscientific, Germany) at 50°C until the constant weight was obtained (Velmurugan and Incharoensakdi, 2018). Intracellular pigments of *Synechocystis* cell suspension were extracted by dimethylformamide. Chlorophyll *a* was determined according to the method of Moran (1982). The polyhydroxybutyrate (PHB) content was determined as described by Monshupanee and Incharoensakdi (2013) using HPLC (Shimadzu, Japan) equipped with InertSustain 3 μm C18 column (GL Sciences, Japan) and UV/Vis detector. The estimation of lipid content was performed using the method described by Monshupanee and Incharoensakdi (2013). The estimation of glycogen was performed by acid hydrolysis followed by sugar analysis by HPLC and the theoretical factor 1.111 was used for the glycogen to glucose conversion (Demirbas, 2005). The sugar and ethanol contents were quantified using HPLC system equipped with refractive index detector (RID 10A, Shimadzu, Japan). Metabolic intermediates such as acetate, pyruvate, and succinate were quantified using HPLC equipped with UV/Vis detector (SPD-20A, Shimadzu, Japan). The components were separated in Phenomenex, Rezex ROA-Organic acid column (150 × 7.8 mm) with 5 mM H_2_SO_4_ as a mobile phase at a flow rate of 0.6 mL/min.

NADP/NADPH from *in*-*vitro* studies and intracellular NADP/NADPH from 25 days grown cells were determined using NADP/NADPH quantification kit (MAK038, SIGMA) according to manufacturer's instruction. Briefly, the metal oxide containing medium was centrifuged (12,000 × g for 5 min) and filtered through syringe filter (0.45 μ) for *in*-*vitro* analysis. For intracellular NADPH/NADP determination, the cells were centrifuged (6,000 × g for 10 min) and the pellet was suspended in phosphate buffered saline (PBS) and centrifuged at 300 × g for 5 min. The pellet was collected and dissolved in extraction buffer and centrifuged at 12,000 × g for 5 min. The supernatant was first filtered with syringe filter (0.45 μ) and again filtered through centrifuging (12,000 × g for 10 min) the samples in MWCO 10 K filtration tube. The filtered samples were used for total NADP (NADP and NADPH) and heated samples (60°C for 30 min) were used for NADPH analysis. Total NADP and NADPH samples were individually quantified at 450 nm on a plate reader (Li et al., 2018).

### Statistical Analysis

All experiments were performed in triplicate and the average values are reported. The average and standard deviation values were calculated using the respective functions (AVERAGE, STDEV) available in Microsoft Excel. The statistical differences were determined according to Duncan's multiple range test at *p* ≤ 0.05. In all figures, different letters on columns indicate a significant difference whereas the same letter indicates no significant difference, with the maximum value starting with the letter a, according to Duncan's multiple range test at *p* ≤ 0.05.

## Results

### *In-vitro* Studies on NADPH Generation

**Figure 1:**
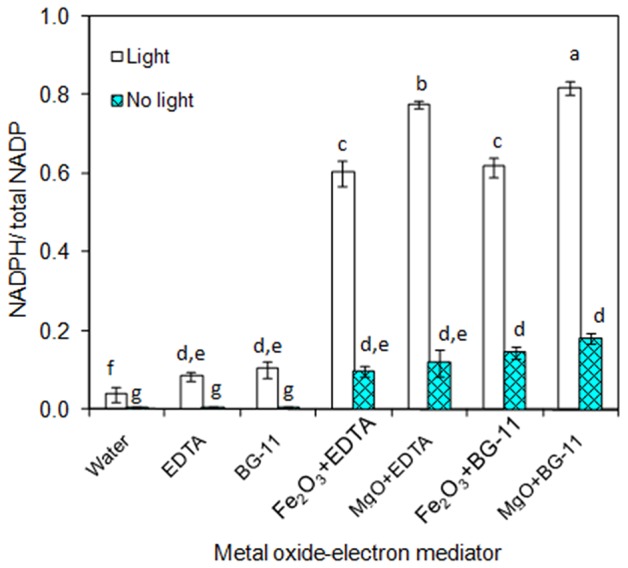
*In-vitro* analysis on influence of metal oxides, electron mediator (EDTA), and light on NADPH regeneration [conditions: water (NADP added), EDTA (3 μM), BG-11 (including 3 μM EDTA), Fe_2_O_3_ (3.2 mg/L), MgO (8 mg/L), NADP (100 μM), and light (100 μE/m^2^/s)]. The letters on each column indicate the significant variation according to Duncan's multiple range test at *p* ≤ 0.05.

As the main purpose of this study relies on the of metal oxide to catalyze the electron transfer from EDTA donor to the NADP acceptor, the individual components such as metal oxides (Fe_2_O_3_ and MgO) and EDTA were characterized for NADPH generation. As can be seen in Figure 1, the 3 μM EDTA and BG-11 media (including 3 μM EDTA) showed almost equal NADPH generation capacity. When comparing the BG-11 medium with water for NADPH generation, the BG-11 medium showed 2-fold improvement. On the other hand, the generation of NADPH was improved to 6- and 8-fold when the metal oxides (Fe_2_O_3_ and MgO) were added, respectively. When comparing the Fe_2_O_3_ and MgO, the MgO showed higher NADPH generation potential. Nevertheless, the NADPH generation was highly reduced in the absence of light. It is worth mentioning that the metal oxide itself generated the NADPH up to a certain level.

### Effect of NADP Concentration on Biomass, Chlorophyll *a*, and Ethanol Production

**Figure 2:**
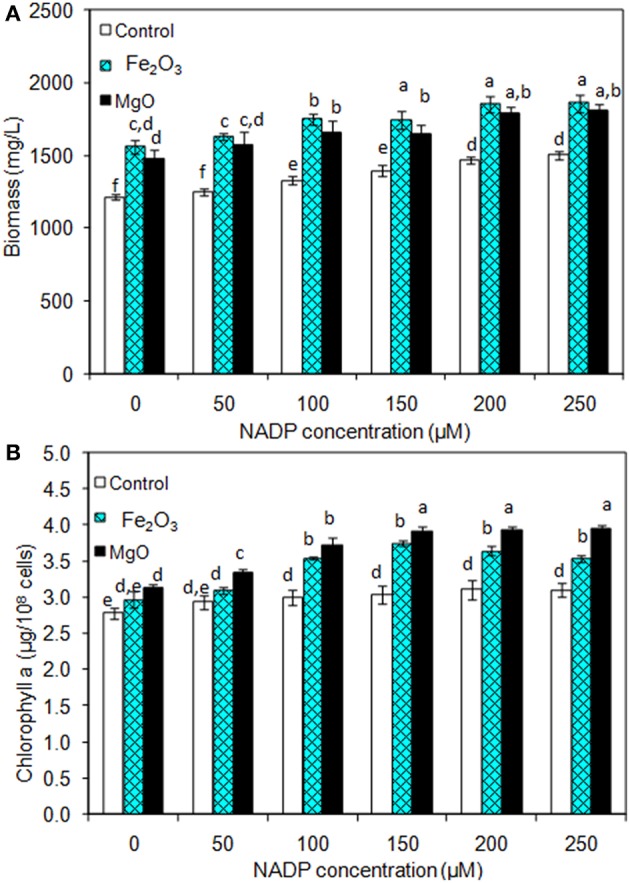
Effect of NADP concentration on **(A)** biomass and **(B)** chlorophyll *a* content under three different conditions [conditions: cultivation time (20 days); control (BG-11); Fe_2_O_3_ (3.2 mg/L), MgO (8 mg/L); and light (100 μE/m^2^/s)]. The letters on each column indicate the significant variation according to Duncan's multiple range test at *p* ≤ 0.05.

As presented in Figures 2A,B, the effect of exogenous NADP on biomass, chlorophyll *a*, and ethanol concentration were analyzed by varying the concentration from 50 to 250 μM. The addition of 50 μM NADP showed no significant improvement in biomass and chlorophyll *a* content; however, when the concentration increased above 100 μM, the contents were increased in all the experiments including the control (BG-11 medium without metal oxide). Similarly, an increase in NADP concentration increased both intracellular and extracellular acetate, succinate, pyruvate, and ethanol concentrations (Table 1). The control experiments showed increased extracellular acetate, succinate, and pyruvate contents, but decreased ethanol content. Upon metal oxide addition, ethanol content was increased whereas other metabolites contents were decreased. The results clearly indicate the redirection of metabolites of very closely related pathways into ethanol synthesis mediated by metal oxides, and the highest ethanol content was observed with 150 μM NADP (Table 1).

**Table 1:** Effect of NADP concentration on intracellular and extracellular products in three different conditions.

**NADP (μM)**	**Intracellular (mg/g)**				**Extracellular (mg/L)**			
	**Acetate**	**Succinate**	**Pyruvate**	**Ethanol**	**Acetate**	**Succinate**	**Pyruvate**	**Ethanol**
**CONTROL (BG-11 MEDIUM WITHOUT METAL OXIDE)**								
0	3.8 ± 0.4^c^	2.9 ± 1.1^c^	4.2 ± 1.1^d^	11.3 ± 1.5^c^	121.2 ± 8.1^c^	116.3 ± 7.6^d^	68.2 ± 9.8^d^	2822.0 ± 245.7^b^
50	4.6 ± 0.8^c^	4.1 ± 0.7^c^	6.7 ± 0.8^c^	13.9 ± 0.9^b^	151.7 ± 10.7^b^	143.5 ± 10.2^c^	94.1 ± 11.7^c^	3076.2 ± 184.1^b^
100	9.5 ± 0.6^b^	8.6 ± 0.6^b^	10.9 ± 0.3^b^	16.4 ± 0.7^a, b^	168.1 ± 11.6^a, b^	169.5 ± 8.1^b^	115.5 ± 11.2^b^	3268.6 ± 214.0^a, b^
150	11.7 ± 1.0^a^	11.0 ± 0.7^a^	13.3 ± 1.0^a^	17.6 ± 1.2^a^	180.6 ± 11.7^a^	186.4 ± 6.5^a^	121.3 ± 9.7^a, b^	3391.0 ± 278.3^a^
200	12.3 ± 0.8^a^	11.4 ± 0.9^a^	13.5 ± 1.1^a^	18.4 ± 1.1^a^	183.7 ± 12.5^a^	192.1 ± 5.9^a^	130.7 ± 10.4^a^	3425.4 ± 302.8^a^
250	12.2 ± 0.9^a^	11.5 ± 1.0^a^	13.4 ± 0.8^a^	18.7 ± 1.6^a^	186.6 ± 10.6^a^	199.1 ± 7.8^a^	135.7 ± 12.2^a^	3511.2 ± 310.4^a^
**Fe_2_O_3_ (3.2 mg/L)**								
0	3.9 ± 1.3^d^	4.2 ± 0.8^d^	5.8 ± 0.8^d^	8.1 ± 0.5^e^	112.3 ± 7.5^c^	111.8 ± 5.4^c^	60.6 ± 7.6^d^	3013.3 ± 235.6^d^
50	5.4 ± 0.4^c^	5.0 ± 0.2^c^	7.4 ± 0.5^c^	10.9 ± 0.7^d^	136.4 ± 10.4^b^	132.1 ± 7.0^b^	84.4 ± 9.2^c^	3521.4 ± 243.7^c^
100	7.9 ± 0.8^b^	7.2 ± 0.4^b^	10.2 ± 0.2^b^	13.6 ± 0.8^c^	148.5 ± 8.6^b^	155.1 ± 5.9^a, b^	107.4 ± 10.0^b^	4138.8 ± 243.0^b^
150	10.6 ± 0.9^a^	9.1 ± 0.9^a^	11.3 ± 0.4^a^	15.7 ± 0.4^b^	162.0 ± 11.3^a^	163.0 ± 5.8^a^	112.1 ± 12.4^a, b^	4721.3 ± 318.4^a^
200	10.3 ± 1.1^a^	9.5 ± 0.7^a^	12.0 ± 0.8^a^	16.9 ± 0.6^a^	165.2 ± 12.8^a^	170.0 ± 6.8^a^	119.1 ± 10.7^a^	4746.8 ± 374.9^a^
250	10.5 ± 0.9^a^	9.8 ± 0.6^a^	12.1 ± 0.7^a^	16.8 ± 0.9^a^	160.2 ± 11.6^a^	172.8 ± 4.6^a^	126.6 ± 11.8^a^	4731.7 ± 365.2^a^
**MgO (8 mg/L)**								
0	3.8 ± 0.5^e^	3.6 ± 0.7^e^	5.1 ± 0.6^e^	7.5 ± 0.6^d^	84.5 ± 8.2^c^	101.9 ± 6.0^e^	43.2 ± 4.7^c^	3752.3 ± 270.5^d^
50	5.5 ± 0.6^d^	5.4 ± 0.3^d^	6.9 ± 0.8^d^	10.2 ± 0.7^c^	98.5 ± 8.7^b^	119.1 ± 5.1^d^	63.5 ± 6.8^b^	4065.6 ± 212.8^c^
100	7.4 ± 1.7^c^	6.9 ± 1.1^c^	8.8 ± 1.1^c^	12.5 ± 0.9^b^	107.4 ± 8.1^b^	135.2 ± 5.6^c^	69.7 ± 7.3^b^	4721.3 ± 198.3^b^
150	11.8 ± 1.1^a^	11.0 ± 0.7^a^	13.3 ± 1.0^a^	17.6 ± 1.5^a^	120.2 ± 16.4^a^	145.0 ± 5.9^b^	78.3 ± 8.2^a, b^	4973.1 ± 282.9^a^
200	9.1 ± 1.2^b^	8.1 ± 0.7^b^	10.7 ± 1.2^b^	16.1 ± 0.5^a^	123.2 ± 7.8^a^	156.9 ± 6.5^a^	84.8 ± 7.6^a^	5028.1 ± 216.0^a^
250	9.4 ± 1.1^b^	8.4 ± 0.9^b^	11.0 ± 0.4^b^	16.4 ± 0.6^a^	123.6 ± 5.5^a^	154.4 ± 10.4^a^	88.2 ± 7.4^a^	5014.7 ± 261.3^a^

*The values are triplicates of three different experiments and the ± values are standard deviation. The superscript letters on each value indicate the significant variation according to Duncan's multiple range test at p ≤ 0.05*.

### Effect of Light Intensity on Biomass, Chlorophyll *a*, and Ethanol Production

**Figure 3:**
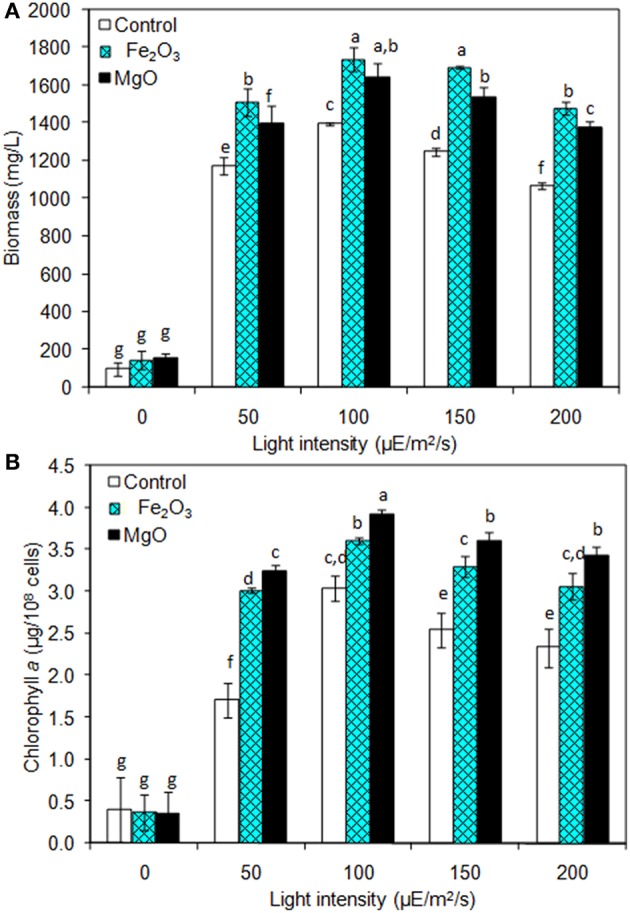
Effect of light intensity on **(A)** biomass and **(B)** chlorophyll *a* content under three different conditions [conditions: cultivation time (20 days), control (BG-11), NADP (150 μM), Fe_2_O_3_ (3.2 mg/L), MgO (8 mg/L), and light (100 μE/m^2^/s)]. The letters on each column indicate the significant variation according to Duncan's multiple range test at *p* ≤ 0.05.

The light is a crucial factor for electron generation in photosynthetic organisms. In this context, we examined the influence of continuous light at various intensities on biomass, chlorophyll *a*, and ethanol production (Figures 3A,B and Table 2). The light highly influences biomass, chlorophyll *a*, and ethanol production, which signifies that the light illumination is an indispensable factor to sustain the growth rate and to regenerate the NADPH (Ma et al., 2017). When comparing the light intensities, the light intensity higher than 100 μE/m^2^/s reduced the biomass, chlorophyll *a* content, and ethanol production. On the other hand, both intracellular and extracellular acetate, succinate, pyruvate, and ethanol contents were significantly increased upon an increase in light intensity upto 100 μE/m^2^/s, above which resulted in the decreased contents (Table 2). It should be noted that even in the presence of externally added metal oxides and NADP, the continuous light did not affect the growth upto normal light illumination (100 μE/m^2^/s), which indicates the compatibility of the integrated treatment in cyanobacterial system (Figure 3A). When comparing the metal oxides, the Fe_2_O_3_ showed higher biomass and chlorophyll *a* content, whereas the MgO produced higher ethanol production (Figures 3A,B and Table 2). Apart from the ethanol production, the other metabolites such as acetate, succinate, and pyruvate were also increased upon Fe_2_O_3_ and MgO addition. The maximum intracellular acetate, succinate, pyruvate, and ethanol contents of 11.8, 11.0, 13.3, and 17.6 mg/g, and the extracellular concentrations of 120.2, 145.0, 78.3, and 4,973.1 mg/L, respectively, were observed upon MgO addition at the light intensity of 100 μE/m^2^/s. Altogether, Fe_2_O_3_ and MgO addition under continuous light intensity of 100 μE/m^2^/s produced highest ethanol concentration of 4,721 and 4,973 mg/L, respectively, in addition to high biomass and chlorophyll *a* contents.

**Table 2:** Effect of light intensity on intracellular and extracellular products in three different conditions.

**Light (μE/m^**2**^/s)**	**Intracellular (mg/g)**				**Extracellular (mg/L)**			
	**Acetate**	**Succinate**	**Pyruvate**	**Ethanol**	**Acetate**	**Succinate**	**Pyruvate**	**Ethanol**
**CONTROL**								
0	1.2 ± 0.3^d^	1.0 ± 0.8^d^	1.4 ± 1.9^d^	1.7 ± 2.0^c^	24.3 ± 4.1^d^	10.3 ± 5.0^e^	7.0 ± 2.8^d^	93.3 ± 11.6^d^
50	7.1 ± 1.4^b^	6.5 ± 1.6^b^	9.8 ± 1.5^b^	12.6 ± 2.3^b^	152.2 ± 9.7^b^	143.8 ± 5.5^b^	105.3 ± 8.0^b^	2,596.7 ± 115.1^b^
100	11.7 ± 1.0^a^	11.0 ± 0.7^a^	13.3 ± 1.0^a^	17.6 ± 1.2^a^	180.6 ± 11.7^a^	186.4 ± 6.5^a^	121.3 ± 9.7^a^	3,391.0 ± 278.3^a^
150	7.4 ± 0.6^b^	6.1 ± 0.3^b^	8.6 ± 1.2^b^	12.2 ± 2.0^b^	149.5 ± 6.5^b^	128.9 ± 5.2^c^	123.9 ± 8.4^a^	2,690.1 ± 104.6^b^
200	5.8 ± 0.4^c^	3.3 ± 0.6^c^	6.4 ± 0.8^c^	9.3 ± 2.4^b^	71.4 ± 6.7^c^	54.1 ± 4.8^d^	42.3 ± 5.7^c^	886.7 ± 169.2^c^
**Fe_2_O_3_ (3.2 mg/L)**								
0	3.4 ± 0.8^d^	2.8 ± 0.6^c^	4.5 ± 1.2^c^	6.1 ± 1.1^c^	29.4 ± 9.6^d^	29.0 ± 5.4^d^	28.9 ± 3.9^d^	164.6 ± 63.0^d^
50	8.9 ± 0.7^b^	8.4 ± 0.3^a^	10.5 ± 1.2^a^	10.7 ± 2.0^b^	114.0 ± 7.7^c^	113.6 ± 8.9^c^	70.2 ± 8.3^c^	3,103.8 ± 149.9^c^
100	10.6 ± 0.9^a^	9.1 ± 0.9^a^	11.3 ± 0.4^a^	15.7 ± 0.4^a^	162.0 ± 11.3^a^	163.0 ± 5.8^a^	112.1 ± 12.4^a^	4,721.3 ± 318.4^a^
150	6.7 ± 0.2^c^	5.8 ± 0.6^b^	7.6 ± 0.5^b^	11.8 ± 0.7^b^	132.3 ± 10.5^b^	134.6 ± 14.2^b^	86.0 ± 6.1^b^	4,534.1 ± 293.0^a^
200	6.3 ± 0.5^c^	5.8 ± 1.1^b^	7.1 ± 1.6^b^	11.7 ± 1.4^b^	122.1 ± 12.7^b, c^	125.2 ± 22.3^b^	79.6 ± 5.5^b, c^	3,762.8 ± 272.3^b^
**MgO (8 mg/L)**								
0	2.9 ± 0.9^c^	2.0 ± 0.8^c^	3.5 ± 1.3^c^	10.0 ± 1.1^d^	29.6 ± 6.2^d^	32.9 ± 6.7^d^	19.5 ± 8.6^d^	195.0 ± 97.1^d^
50	10.2 ± 1.2^a^	9.3 ± 1.8^a^	11.4 ± 2.0^a^	13.7 ± 2.5^b, c^	83.2 ± 5.3^c^	102.9 ± 6.2^c^	50.6 ± 4.9^c^	3,243.4 ± 270.2^c^
100	11.8 ± 1.1^a^	11.0 ± 0.7^a^	13.3 ± 1.0^a^	17.6 ± 1.5^a^	120.2 ± 16.4^a^	145.0 ± 5.9^a^	78.3 ± 8.2^a^	4,973.1 ± 282.9^a^
150	5.0 ± 1.0^b^	4.1 ± 0.6^b^	6.3 ± 0.6^b^	14.9 ± 1.3^b^	106.6 ± 12.0^b^	128.1 ± 5.1^b^	63.7 ± 3.7^b^	4,908.5 ± 224.5^a^
200	4.7 ± 0.7^b^	3.7 ± 0.4^b^	5.6 ± 0.8^b^	12.0 ± 0.9^c^	99.3 ± 10.7^b^	120.5 ± 6.0^b^	57.5 ± 3.5^c^	4,132.6 ± 225.4^b^

*The values are triplicates of three different experiments and the ± values are standard deviation. The superscript letters on each value indicate the significant variation according to Duncan's multiple range test at p ≤ 0.05*.

### NADPH Regeneration on *Synechocystis* and Ethanol Synthesis

**Figure 4:**
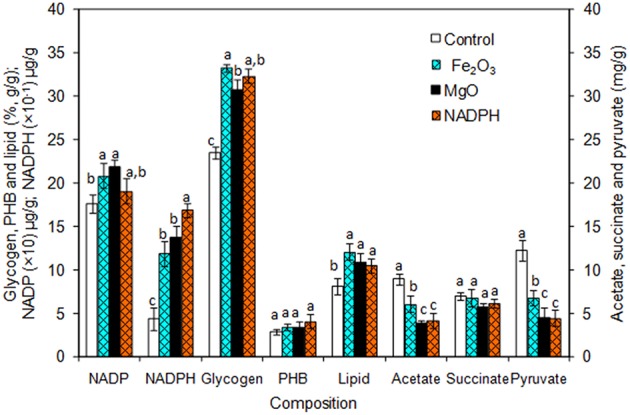
Composition of intracellular products of *Synechocystis* [conditions: cultivation time (25 days) control (BG-11), Fe_2_O_3_ (3.2 mg/L), MgO (8 mg/L), NADP (150 μM), and light (100 μE/m^2^/s)]. The letters on each column indicate the significant variation according to Duncan's multiple range test at *p* ≤ 0.05.

**Figure 5:**
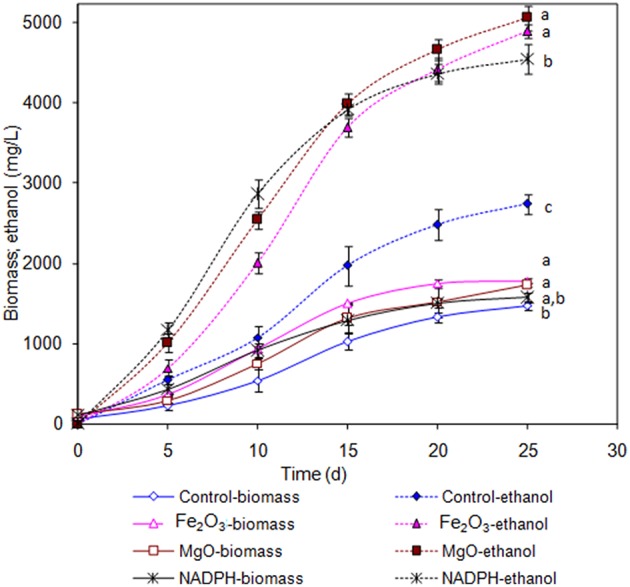
Effect of incubation time on ethanol and biomass concentration [conditions: control (BG-11) Fe_2_O_3_ (3.2 mg/L), MgO (8 mg/L), NADP (150 μM), and light (100 μE/m^2^/s)]. The letters on each point indicate the significant variation according to Duncan's multiple range test at *p* ≤ 0.05.

The *Synechocystis* was cultivated at 5 L level to analyse the feasibility of NADPH regeneration at large scale. The intracellular NADP content in control was 176.1 μg/g DCW, while it was increased to 208.3 and 218.1 μg/g DCW in Fe_2_O_3_ and MgO treated cultures, respectively. As the *Synechocystis* itself has the NADPH regeneration mechanism inside the cell, 0.43 μg/g DCW of NADPH content was observed in control (without metal oxides), whereas it was increased to 1.18 and 1.38 μg/g DCW in Fe_2_O_3_ and MgO treated cultures, which is 2.74- and 3.16-fold higher than the control, respectively (Figure 4). On the other hand, the NADPH supplemented experiment showed higher intracellular NADPH and NADP contents than the control. The intracellular glycogen, PHB, and lipid contents in *Synechocystis* were analyzed to monitor the changes in metabolism of engineered *Synechocystis* at 5 L level (Figure 4). After 25 days of growth, the engineered strain produced 23.8% (g/g DCW) glycogen in BG-11 medium containing 150 μM NADP, whereas it was increased upon the addition of Fe_2_O_3_ and MgO. The maximum glycogen content of 33.2% was observed with Fe_2_O_3_, whereas MgO produced 30.7%. The PHB content was slightly increased upon Fe_2_O_3_ and MgO addition. Similar to the results on glycogen content, the lipid content was also increased with Fe_2_O_3_ and MgO, with Fe_2_O_3_ having the maximum lipid content. The glycogen, PHB, and lipid contents in control experiments were lower than those of metal oxide treated experiments, which indicated that the metal oxide mediated NADPH regeneration contributed to the increase of glycogen, PHB, and lipid accumulation (Figure 4). The 1.5-fold increase in lipid content upon NADPH regeneration was already reported by Osada et al. (2017). The results further confirm that the presence of metal oxides regenerated the NADPH and improved the lipid accumulation. The analysis of intracellular metabolites such as acetate, succinate, and pyruvate showed the significant improvement in their contents upon NADPH regeneration (Figure 4). The highest intracellular acetate, succinate, and pyruvate contents of about 8.9, 7.0, and 12.2 mg/g, respectively, were observed in the control experiments. The ethanol and biomass production was increased with an increase in incubation time and reached the maximum at 25 days (Figure 5). When comparing the effect of Fe_2_O_3_ and MgO on ethanol production, the MgO showed higher ethanol production (Figure 5). The NADPH supplemented experiments initially showed (up to 10 days) higher ethanol concentration, after which the production was decreased when compared to the metal oxide added culture. When comparing the ethanol concentration of 5 L level experiments at 20 days, the ethanol concentration was slightly lower than that observed in flask experiments. The maximum ethanol concentrations of 4,851 and 5,100 mg/L were observed with Fe_2_O_3_ and MgO treatments at 25 days, respectively.

## Discussion

### NADPH Is Generated in the Presence of Metal and EDTA

The chemical method for NADPH generation was performed by adding metal oxides (Fe_2_O_3_ or MgO) in BG-11 medium containing the EDTA. In the presence of EDTA, the Fe_2_O_3_, and MgO were partially solubilized, even though it has slow dissolution in water (Jones et al., 2015). Similar to the present study, regeneration of NADH in the absence of enzyme was improved using the metal oxides such as carbon containing TiO_2_ and P-doped TiO_2_ (Jiang et al., 2005; Shi et al., 2006). According to Jiang et al. (2005), the exposure of metal oxide (Fe_2_O_3_ or MgO) to light illumination generates electron. However, the present study demonstrated that the EDTA can also donate the electron to efficiently reduce the NADP to NADPH in the presence of metal oxides. Furthermore, those metal oxides reported for cofactor regeneration may also inhibit the growth of cyanobacteria (Comotto et al., 2014; Xiong et al., 2017).

### Exogenous NADP Improves NADPH Regeneration and Ethanol Production

The addition of NADP improved the biomass and ethanol concentrations even without metal oxide addition (control), which suggest that the metal oxide does not contribute to the growth under NADP supplemented condition. However, the metal oxides facilitate the reduction of added NADP into NADPH, which is utilized for increased ethanol production (Figures 2A,B and Table 1). Recently, an attempt to engineer glucose-6-phosphate dehydrogenase gene (pentose phosphate pathway) in *Synechocystis* sp. PCC 6803 to enhance the reduction of NADP into NADPH was successful in improving the ethanol production (Choi and Park, 2016). In the present study, the increased NADPH regeneration mediated by metal oxides has been achieved without engineering other natural pathways of *Synechocystis* and the production of ethanol was also higher than that reported by Choi and Park (2016). The increase in acetate, pyruvate, succinate, and ethanol concentration in control experiment (BG11 medium without metal oxides) clearly indicates that the use of NADP to regenerate NADPH does not only improve the biomass and chlorophyll *a* content, but also enhances the overall metabolism (Table 1).

### Optimum Light Intensity Improves Biomass, Chlorophyll *a* Content, and Ethanol Synthesis

As the NADPH regeneration process combines chemical and microbial studies, light intensity was optimized compatible for microbial growth and ethanol production (Figure 3 and Table 2). The decrease in cell growth and chlorophyll *a* content at high light intensities might be due to the reduced content of photosynthetic apparatus. Kopecná et al. (2012) investigated the influence of low (10 and 40 μE/m^2^/s) and high light intensity (150 and 300 μE/m^2^/s) on *Synechocystis* sp. PCC 6803 growth and chlorophyll *a* content. They found that low light facilitated the PSI generation, while high light reduced the growth and chlorophyll content by decreasing the PSI. In the present study, favorable light intensity was at 100 μE/m^2^/s even in the absence of metal oxides (control). The addition of metal oxides was always beneficial with respect to biomass and chlorophyll *a* content in the presence of light, while in the absence of light the metal oxides showed no significant effect (Figures 3A,B). Similarly, the light intensity up to 100 μE/m^2^/s increased the metabolites such as acetate, succinate, and pyruvate intracellularly and extracellularly under three different conditions (control, Fe_2_O_3_m, and MgO), and negative effect was observed at light intensity higher than 100 μE/m^2^/s (Table 2). The changes in metabolite contents as affected by light intensity correlated with the changes in biomass and chlorophyll *a* content (Figures 3A,B and Table 2).

For ethanol, light intensity at 100 μE/m^2^/s showed no changes in intracellular ethanol content, with a maximum value of 17.6 mg/g, either with or without metal oxides addition, while extracellular ethanol concentration was increased upon metal oxides addition (Table 2). This suggested the excretion of ethanol by the cells when their ethanol content reached a certain level. The maximum extracellular ethanol concentration at 100 μE/m^2^/s indicates the importance of light intensity on improving the ethanol production through NADPH regeneration.

### NADPH Regeneration Redirects the Carbon Flow Toward Ethanol Synthesis

An increase in NADPH upon metal oxide addition indicates the successful regeneration of NADPH. The metal oxide mediated extracellular NADPH regeneration produced higher ethanol and biomass contents than those by direct supplementation of NADPH (Figure 5). The increase in NADPH might be due to direct transfer of NADPH through specific transport system or transfer through permeability transition pore as reported previously (Lu et al., 2007; Pittelli et al., 2011; Xiao et al., 2018). Further work is required to study the mechanism of transport of extracellular NADPH across the plasma membrane of cyanobacteria. NADPH is considered as the universal electron donor for reductive biosynthetic process, it also plays a role in detoxification of the cell. With respect to ethanol biosynthesis, the stress imposed by increased ethanol level might lead to cellular redox imbalance and a NADP shortage (Chandler et al., 2004; Valadi et al., 2004; You et al., 2015). The supply of external NADPH would mitigate the redox imbalance to maintain the NADPH dependent biosynthetic process of various products including ethanol. Another beneficial aspect of an increased NADPH might be its contribution as cofactor for the glyceraldehyde-3-phosphate dehydrogenase, which was reported to be upregulated upon ethanol stress in yeast (Alexandre et al., 2001).

The results clearly indicate the proper distribution of metal oxides (Fe_2_O_3_ and MgO), EDTA, and NADP are important for an optimal regeneration of NADPH in *Synechocystis* at large scale. The successful use of light illumination for co-factor regeneration has been previously reported which utilizes microorganisms as a source of the enzyme oxidoreductases (Nakamura and Yamanaka, 2002; Ma et al., 2017). The alcohol dehydrogenase engineered *Rhodobacter sphaeroides* could regenerate the co-factor efficiently in the presence of light during the production of chlorophenyl ethanol (Ma et al., 2017). However, the present study showed for the first time the production of ethanol integrated with metal oxide mediated NADPH regeneration.

Apart from the extracellular release of metabolites, the evaporation of volatile component including ethanol is also possible. Woods et al. (2012) used a closed type photobioreactor to recover the evaporated ethanol under sun light and reported that the ethanol obtained from condensate (272.41 mM) was always higher than the ethanol obtained from the medium (233.75 mM). The ethanol concentration obtained from the present study is 5,100 mg/L (≈115 mM) and the use of a closed photobioreactor to recover the evaporated ethanol content can be beneficial to obtain more ethanol yield. The *in-vitro* studies and NADPH regeneration in *Synechocystis* culture clearly demonstrate that the metal nanoparticles have the potential to regenerate the NADPH and improve the ethanol production. The carbon flux in engineered *Synechocystis* metabolism indicates that the NADPH regeneration potentially redirects the carbon from very closely related pathway into ethanol synthesis pathway.

## Conclusion

As the cyanobacterial ethanol production involves phototrophic growth condition, the use of light energy for NADPH regeneration is feasible. However, the light energy alone has no potential to obtain an effective regeneration process. In these aspects, the metal oxides were applied in both *in-vitro* as well as microbial experiments. The addition of metal oxides such as Fe_2_O_3_ and MgO significantly improved the NADPH regeneration, which further improved the ethanol production upto 5,100 mg/L at 5 L scale. Thus, we demonstrated that introducing metal oxide mediated NADPH regeneration is a promising strategy to increase the ethanol production in engineered *Synechocystis* under photoautotrophic growth condition.

## Data Availability

No datasets were generated or analyzed for this study.

## Author Contributions

AI and RV participated in designing of work, interpretation of data, and writing of the manuscript. RV performed the experiments.

### Conflict of Interest Statement

The authors declare that the research was conducted in the absence of any commercial or financial relationships that could be construed as a potential conflict of interest.

## References

[B1] Alexandre H. Ansanay-Galeote V. Dequin S. Blondin B. (2001). Global gene expression during short-term ethanol stress in *Saccharomyces cerevisiae*. FEBS Lett. 498, 98–103. 10.1016/S0014-5793(01)02503-0 11389906

[B2] Asahi R. Morikawa T. Ohwaki T. Aoki K. Taga Y. (2001). Visible-light photocatalysis in nitrogen-doped titanium oxides. Science 293, 269–271. 10.1126/science.1061051 11452117

[B3] Chandler M. Stanley G. A. Rogers P. Chambers P. (2004). A genomic approach to defining the ethanol stress response in the yeast *Saccharomyces cerevisiae*. Ann. Microbiol. 54, 427–454.

[B4] Choi Y. N. Park J. M. (2016). Enhancing biomass and ethanol production by increasing NADPH production in *Synechocystis* sp. PCC 6803. Bioresour. Technol. 213, 54–57. 10.1016/j.biortech.2016.02.056 26951740

[B5] Comotto M. Casazza A. A. Aliakbarian B. Caratto V. Ferretti M. Perego P. (2014). Influence of TiO_2_ nanoparticles on growth and phenolic production in photosynthetic microorganisms. Sci. World J. 2014:961437. 10.1155/2014/961437 25610914 PMC4291162

[B6] Demirbas A. (2005). Bioethanol from cellulosic materials: a renewable motor fuel from biomass. Energy Sour. 27, 327–337. 10.1080/00908310390266643

[B7] Englund E. Andersen-Ranberg J. Miao R. Hamberger B. Lindberg P. (2015). Metabolic engineering of *Synechocystis* sp. PCC 6803 for production of the plant diterpenoidmanoyl oxide. ACS Synth. Biol. 4, 1270–1278. 10.1021/acssynbio.5b00070 26133196 PMC4685428

[B8] Gao Z. Zhao H. Li Z. Tan X. Lu X. (2012). Photosynthetic production of ethanol from carbon dioxide in genetically engineered cyanobacteria. Energy Environ. Sci. 5, 9857–9865. 10.1039/C2EE22675H

[B9] Grimes M. T. Drueckhammer D. G. (1993). Membrane-enclosed electroenzymatic catalysis with a low molecular weight electron transfer mediator. J. Org. Chem. 58, 6148–6150. 10.1021/jo00074a056

[B10] Jiang Z. Lu C. Wu H. (2005). Photoregeneration of NADH using carbon containing TiO_2_. Ind. Eng. Chem. Res. 44, 4165–4170. 10.1021/ie049155w

[B11] Johannes T. W. Woodyer R. D. Zhao H. (2007). Efficient regeneration of NADPH using an engineered phosphite dehydrogenase. Biotechnol. Bioeng. 96, 18–26. 10.1002/bit.21168 16948172

[B12] Jones A. M. Griffin P. J. Waite T. D. (2015). Ferrous iron oxidation by molecular oxygen under acidic conditions: the effect of citrate, EDTA and fulvic acid. Geochim. Cosmochim. Acta 160, 117–131. 10.1016/j.gca.2015.03.026

[B13] Kanwal S. Rastogi R. P. Incharoensakdi A. (2014). Glutamate decarboxylase activity and gamma-aminobutyric acid content in *Synechocystis* sp. PCC 6803 under osmotic stress and different carbon sources. J. Appl. Phycol. 26, 2327–2333. 10.1007/s10811-014-0259-9

[B14] Kopecná J. Komeda J. Bucinská L. Sobotka R. (2012). Long-term acclimation of the cyanobacterium *Synechocystis* sp. PCC 6803 to high light is accompanied by an enhanced production of chlorophyll that is preferentially channeled to trimeric photosystem I. Plant Physiol. 160, 2239–2250. 10.1104/pp.112.207274 23037506 PMC3510144

[B15] Li W. Liu S. Zhang M. Zhao H.-P. Zheng P. (2018). Oxidation of organic electron donor by denitratation: Performance, pathway and key microorganism. Chem. Eng. J. 343, 554–560. 10.1016/j.cej.2018.02.112

[B16] Liang F. Englund E. Lindberg P. Lindblad P. (2018). Engineered cyanobacteria with enhanced growth show increased ethanol production and higher biofuel to biomass ratio. Metab. Eng. 46, 51–59. 10.1016/j.ymben.2018.02.006 29477858

[B17] Lu H. Burns D. Garnier P. Wei G. Zhu K. Ying W. (2007). P2X7 receptors mediate NADH transport across the plasma membranes of astrocytes. Biochem. Biophys. Res. Commun. 362, 946–950. 10.1016/j.bbrc.2007.08.095 17803959

[B18] Ma X. Liang H. Ning C. Deng S. Su E. (2017). Integrating a light-driven coenzyme regeneration system by expression of an alcohol dehydrogenase in phototrophic bacteria for synthesis of chiral alcohol. J. Biotechnol. 259, 120–125. 10.1016/j.jbiotec.2017.07.032 28760442

[B19] Monshupanee T. Incharoensakdi A. (2013). Enhanced accumulation of glycogen, lipids and polyhydroxybutyrate under optimal nutrients and light intensities in the *Cyanobacterium synechocystis* sp. Pcc 6803. J. Appl. Microbiol. 116, 830–838. 10.1111/jam.12409 24299499

[B20] Moran R. (1982). Formulae for determination of chlorophyllous pigments extracted with *N,N* dimethyl formamide. Plant Physiol. 69, 1376–1381. 10.1104/pp.69.6.1376 16662407 PMC426422

[B21] Motekaitis R. Martell A. E. Hayes D. (1980). The Iron (III)-catalyzed oxidation of edta in aqueous solution. Can. J. Chem. 58, 1999–2005. 10.1139/v80-318

[B22] Nakamura K. Yamanaka R. (2002). Light mediated cofactor recycling system in biocatalytic asymmetric reduction of ketone. Chem. Commun. 16, 1782–1783. 10.1039/b203844g 12196997

[B23] Nozzi N. E. Oliver J. W. Atsumi S. (2013). Cyanobacteria as a platform for biofuel production. Bioeng. Biotechnol. 1:7. 10.3389/fbioe.2013.00007 25022311 PMC4090892

[B24] Osada K. Maeda Y. Yoshino T. Nojima D. Bowler C. Tanaka T. (2017). Enhanced NADPH production in the pentose phosphate pathway accelerates lipid accumulation in the oleaginous diatom *Fistulifera solaris*. Algal Res. 23, 126–134. 10.1016/j.algal.2017.01.015

[B25] Pittelli M. Felici R. Pitozzi V. Giovannelli L. Bigagli E. Cialdai F.. (2011). Pharmacological effects of exogenous NAD on mitochondrial bioenergetics, DNA repair, and apoptosis. Mol. Pharmacol. 80, 1136–1146. 10.1124/mol.111.073916 21917911

[B26] Rippka R. Deruelles J. Waterbury J. B. Herdman M. Stanier R. Y. (1979). Generic assignments, strain histories and properties of pure cultures of cyanobacteria. J. Gen. Microbiol. 111, 1–61. 10.1099/00221287-111-1-1

[B27] Shi Q. Yang D. Jiang Z. Li J. (2006).Visible-light photocatalytic regeneration of NADH using P-doped TiO_2_ nanoparticles. J. Mol. Catal. B Enzym. 43, 44–48. 10.1016/j.molcatb.2006.06.005

[B28] Valadi H. Valadi A. Ansell R. Gustafsson L. Adler L. Norbeck J. (2004). NADH-reductive stress in *Saccharomyces cerevisiae* induces the expression of the minor isoform of glyceraldehydes-3-phosphate dehydrogenase (TDH1). Curr. Genet. 45, 90–95. 10.1007/s00294-003-0469-1 14652693

[B29] van der Donk W. A. Zhao H. (2003). Recent developments in pyridine nucleotide regeneration. Curr. Opin. Biotechnol. 14, 421–426. 10.1016/S0958-1669(03)00094-6 12943852

[B30] Velmurugan R. Incharoensakdi A. (2016). Potential of metal oxides in fractionation of *Synechocystis* sp. PCC 6803 biomass for biofuel production. Algal. Res. 19, 96–103. 10.1016/j.algal.2016.07.018

[B31] Velmurugan R. Incharoensakdi A. (2018). Disruption of polyhydroxybutyrate synthesis redirects carbon flow towards glycogen synthesis in *Synechocystis* sp. Pcc 6803 overexpressing *Glgc*/*Glga*. Plant Cell Physiol. 59, 2020–2029. 10.1093/pcp/pcy121 29931091

[B32] Wong C.-H. Drueckhammer D. G. Sweers H. M. (1985). Enzymatic vs. fermentative synthesis: Thermostable glucose dehydrogenase catalyzed regeneration of NAD(P)H for use in enzymatic synthesis. J. Am. Chem. Soc. 107, 4028–4031. 10.1021/ja00299a044

[B33] Woods R. P. Malkiel E. Moll B. Legere E. (2012). Closed Photobioreactor System for Continued Daily in situ Production of Ethanol From Genetically Enhanced Photosynthetic Organisms With Means for Separation and Removal of Ethanol. US Patent No. 8586353B2. Washington, DC: U.S. Patent and Trademark Office.

[B34] Xiao W. Wang R.-S. Handy D. E. Loscalzo J. (2018). NAD(H) and NADP(H) redox couples and cellular energy metabolism. Antioxid. Redox. Signal. 28, 251–272. 10.1089/ars.2017.7216 28648096 PMC5737637

[B35] Xiong W. Tang Y. Shao C. Zhao Y. Jin B. Huang T.. (2017). Prevention of cyanobacterial blooms using nanosilica: a biomineralization-inspired strategy. Environ. Sci. Technol. 51, 12717–12726. 10.1021/acs.est.7b02985 28949533

[B36] Yamamoto H. Mitsuhashi K. Kimoto N. Kobayashi Y. Esaki N. (2005). Robust NADH-regenerator: improved α-haloketone-resistant formate dehydrogenase. Appl. Microbiol. Biotechnol. 67, 33–39. 10.1007/s00253-004-1728-x 15338080

[B37] You L. He L. Tang Y. J. (2015). The photoheterotrophic fluxome in *Synechocystis* sp. PCC 6803 and its implications for cyanobacterial bioenergetics. J. Bacteriol. 197, 943–950. 10.1128/JB.02149-14 25535269 PMC4325091

[B38] Zhu Z. Luan G. Tan X. Zhang H. Lu X. (2017). Rescuing ethanol photosynthetic production of cyanobacteria sterilized outdoor cultivations with a bicarbonate-based pH-rising strategy. Biotechnol. Biofuels 10:93. 10.1186/s13068-017-0765-5 28416967 PMC5391583

