# Supplementary material for: Metal Oxide Mediated Extracellular NADPH Regeneration Improves Ethanol Production by Engineered Synechocystis sp. PCC 6803
Source: Front Bioeng Biotechnol. 2019 Jun 19;7:148. doi: 10.3389/fbioe.2019.00148 (PMC6593046; doi:10.3389/fbioe.2019.00148)
Supplement: Supplementary file 1 [file Table_1.DOC]

**Data S1 Construction of Ethanol Synthesis pathway into *Synechocystis***

Taq polymerase, ligase and restriction enzymes (XbaI, SpeI and Pst I) were purchased from Fermentas (Canada). The kits used for plasmid extraction and PCR purification were obtained from Geneaid Biotech Ltd. Oligonucleotide primers were designed with the help of primer3 online software and the synthesis of nucleotide sequences was performed by Pacific Science Co. Ltd, Thailand. The pEERM vector is a kind gift from Professor Peter Lindblad, Uppsala University, Sweden.

The expression vector pAPX was constructed by insertion of alcohol dehydrogenase (*adh*: slr0942) into pEERM vector under the control of *psbA2* promoter. The gene *adh* was amplified with forward (ACTAGTGTGCAGAGTTTCAATAGG) and reverse (CTGCAGTTAAATTTCATCCCATAGG) primers using the genomic DNA of *Synechocystis* as a template and the gene *pdc* was with forward (GCGTCTAGAATGTCTGAAATTACTTTGG) and reverse (GCACTAGTTTATTGCTTAGCGTTGGT) primers using the genomic DNA of *S. cerevisiae* as a template.

*Escherichia coli* DH5α strain was used for routine propagation of plasmid constructs and the plasmid construct was transformed into the *Synechocystis*sp.PCC 6803 as outlined in previous study (Velmurugan and Incharoensakdi, 2018). After the transformation, the successful insertion of *pdc* and *adh* genes into the *PSBA2* site of the *Synechocystis* sp. PCC 6803 genome was confirmed by PCR using *PSBA2* forward (TAGCCCTTTTCCAACAACAAT) and reverse (TGTAGCCATTACAAATGATTAGGG) primers.
